# Beyond competence: advance directives in dementia research

**DOI:** 10.1007/s40592-015-0034-y

**Published:** 2015-10-12

**Authors:** Karin Rolanda Jongsma, Suzanne van de Vathorst

**Affiliations:** Department of Medical Ethics and Philosophy of Medicine, Erasmus University Medical Centre Rotterdam, Office NA 21.17, PO box 2040, 3000 CA Rotterdam, The Netherlands

**Keywords:** Dementia, Informed consent, Advance directives, Research ethics

## Abstract

Dementia is highly prevalent and incurable. The participation of dementia patients in clinical research is indispensable if we want to find an effective treatment for dementia. However, one of the primary challenges in dementia research is the patients’ gradual loss of the capacity to consent. Patients with dementia are characterized by the fact that, at an earlier stage of their life, they were able to give their consent to participation in research. Therefore, the phase when patients are still competent to decide offers a valuable opportunity to authorize research, by using an advance research directive (ARD). Yet, the use of ARDs as an authorization for research participation remains controversial. In this paper we discuss the role of autonomous decision-making and the protection of incompetent research subjects. We will show why ARDs are a morally defensible basis for the inclusion of this population in biomedical research and that the use of ARDs is compatible with the protection of incompetent research subjects.

## Introduction

Imagine this typical, and unfortunately prevalent, case: Mr. Jansen has been suffering from dementia^1^ for the past 4 years. At the moment there is no cure for dementia and he will lose more and more of his mental capacities. While previously in his life he was able to make his own decisions, he is now at the point where he has lost the capacities needed to competently make decisions. His wife will now have to make decisions for him. One of the decisions she is facing is whether he will participate in a research trial that aims to develop new treatments for the cognitive decline due to dementia.

The participation in clinical research of dementia patients like Mr. Jansen is essential for the development of more effective diagnostic instruments and therapeutic interventions for this condition (Downs 1997; Warner and Nomani 2008; Selkoe 1992). Even though the inclusion of dementia patients in research is necessary, it is also important that their participation is based on appropriate consent regimes. Patients with dementia face an increased risk of becoming incompetent to provide informed consent. In most countries, the legal possibilities for doing research with incapacitated research subjects are limited^2^ and require the consent of a legal representative (European Clinical Trials Directive 2001; Biomedicine Convention 1997). These measures also have clear downsides: they restrict the possibilities of doing research with dementia patients, are not based upon autonomous authorization of the research participant and decisions made by legal representatives do not necessarily conform to what the patients choose whilst still competent (e.g. Stocking et al. 2006). Consent by a legal representative has therefore been criticized for failing to represent the patient’s wishes (Shalowitz et al. 2006). Given the often slowly progressive nature of dementia, and the possibility of anticipating future incompetence, we suggest allowing dementia patients to anticipate future research participation by signing an advance research directive (ARD). In an ARD the dementia patient could describe his preferences concerning future research participation.

ARDs are not yet commonly used in practice. In the United States, even though it is not a legal standard, the NIH allows dementia patients to use ARDs, but these seem to have a low rate of completion (Muthappan et al. 2005). In Europe, ARDs are not yet used in the context of research,^3^ but for many health-care decisions, such as organ donation and end-of-life decisions, advance directives are accepted and widely used. The use of advance directives in research remains, nevertheless, controversial, it is argued for example that legal representatives should make decisions based on the concept of “best-interest” and that an anticipated decision is neither free nor informed (Dresser 2001, 2014; Fagerlin and Schneider 2004).

In this paper we address the following questions: (1) do ARDs provide a morally defensible basis for including incapacitated dementia patients in research trials, (2) are ARDs a better alternative to research authorization than consent by a legal representative? and (3) what are the problems raised by the use of ARDs in dementia research? We will argue that ARDs are a valuable authorization tool, provide a morally defensible basis for the inclusion of dementia patients in research, and are the better alternative to consent by a legal representative. Furthermore we will show that the remaining issues all have to do with protection during the trial and with withdrawal. Therefore we will start by discussing the moral aims of informed consent as the gold standard for research authorization, and then we explain the problems concerning the current practice of consent by a legal representative and describe why ARDs are a reasonable option for research authorization. Finally, we will discuss controversies for using ARDs, and investigate possible solutions.

## The moral aims of informed consent

The gold standard for acquiring a patient’s permission to be included in research is “informed consent”. The informed-consent requirement gained prominence in reaction to abuse of people in various experiments. *The Nuremberg Code* of research ethics, an influential response to the cruelty of Nazi experiments, stipulated: “the voluntary consent of the human subject is absolutely essential” (The Nuremberg Code 1949). The research participant must be *adequately* informed about relevant facts of the research trial, and must provide free and informed consent (The Nuremberg Code 1949; Beauchamp and Childress 2013). The moral base of informed consent in research lies in the ethical obligation of respect for persons. Respect for persons requires us to acknowledge the value of other persons and to treat them as ends in themselves and not merely as means to ends (Belmont Report 1979). Respect for persons has two moral dimensions: respect for autonomy and protection of persons with diminished autonomy. Respect for autonomy derives from the recognition that persons are rational and reflective beings who can choose to live according to their values. Values are developed and learned over a time period, adjusted, reflected upon and embodied. From these values, lasting orientations with a rational component are derived, which shape the preferences of the agent. This in contrast to wishes, which are merely an emotional desire at a specific point in time and may change more rapidly. Wishes are not necessarily in concordance with one’s values. When a wish and a preference are not concordant, an autonomous agent can decide whether his preferences and values are primary, or whether he follows a wish.

Decisions of autonomous persons should be respected even when these do not serve the well-being or the best interest of the person in an objective sense; it is his life, it belongs to him and no one else (Feinberg 1989). Acts that could harm or seem wrongful can be considered rightful as long as a person voluntarily consents to bear these adverse consequences. As John Harris said: “consent is a dimension of respect for persons in that it is through consenting to things that affect us that we make those things consistent with our own values. When we consent to what others propose we make their ends and objectives part of our own plans” (Harris 2003). As biomedical research is not primarily aimed at the wellbeing of the participant—its primary aim is to collect data and gain knowledge—autonomous authorization is absolutely necessary. This authorization is generally understood as a continuous process; throughout the research trial the patient should be willing to continue participation, and is free to withdraw at any time for any reason.

The informed-consent approach respects the autonomy of participants competent to make decisions, but is problematic when subjects lack decision-making capacity. Dementia patients progressively lose higher cognitive functions such as memory, reasoning, comprehension and judgment and understanding in the more advanced stages (Bielby 2008; Logsdon et al. 2002; McKhann et al. 2011). Therefore, they face an increased risk of becoming incompetent.^4^ When a person becomes incompetent and is not (fully) capable of providing free, voluntary and informed consent, the second meaning of respect for persons acquires prominence protection. The extent of the required protection varies for the capacities that are lost, depends on the specific situation and should depend on the risk of harm and the likelihood of benefit (Belmont Report 1979). The protection of incompetent research subjects is not absolute; it does not result in the exclusion of incompetent persons from research altogether, but additional requirements regarding the risk and burden of research apply in most jurisdictions and the consent of a legal representative is required. The protection of incompetent research subjects should, however, not imply the complete exclusion of the incompetent person from the authorization process, especially not when this person anticipated his future incompetence.

## The problems of consent by a legal representative

In the light of the moral aims of the informed consent requirement it becomes apparent that the current legal standard of substituted authorization by a legal representative (proxy consent) is problematic in several ways. In this section we will argue that (1) proxy consent does not do justice to the voice of the research participant, (2) making decisions as a legal representative is no easy task and (3) research possibilities are limited when autonomous authorization by the research subject is not possible.

Firstly, the authority of proxy consent comes from the assumption that legal representatives know the incapacitated person well and can give voice to what the research participants would have decided. This is also reflected in the fact that some legal guidelines for proxy consent require the legal representative to act on the basis of the persons’ presumed will (e.g. WMO Dutch National Law, European Clinical Trials Directive 2001). However, even with good intentions and with knowing each other well, epistemic problems persist due to the lack of transparency we have to each other, especially in unusual situations (Holm 2001). The underlying assumption that legal representatives know what the incapacitated person would have decided is thus questionable, and the proxies’ judgements about their loved ones’ preferences are often discordant (Kim et al. 2013). Empirical studies in which legal representatives and patients with mild dementia were interviewed separately about the willingness to participate in clinical research trials show that legal representatives are either too reluctant to authorize enrolment in clinical trials, or consent to studies that do not really correspond to the preferences and values of the persons they represent (e.g. Stocking et al. 2006; Shalowitz et al. 2006). Decisions made by legal representatives do not respect the autonomy of research participants, because they do little justice to the preferences of incapacitated subjects. Therefore they are a poor means to extend the incompetent participant’s voice in the decision-making process. Interestingly, a majority of dementia patients would leave their legal representative (some) leeway to make decisions for them in the future that differ from their own preferences, if the legal representative would get access to better or more information (Kim et al. 2013). However, a minority would not be comfortable with legal representatives making decisions against their own preferences (Kim et al. 2013; Stocking et al. 2006), these persons would want their legal representatives to give voice to their own preferences and wishes.

Secondly, legal representatives experience their task to make decisions for their incompetent loved ones’ as difficult, and have problems in bearing the burden and responsibility of making decisions for a dementia patient (Livingston 2010; Sugarman et al. 2001). They experience guilt and stress and have problems with processing the provided information (Wendler and Rid 2011). To make decisions as a legal representative is especially hard in circumstances in which long time roles and patterns of authority are reversed and confidences are sometimes breached (Livingston et al. 2010). For example, it is conceivable that a child who has always been obedient to his parents, will have difficulties in taking the lead when his authoritarian parent becomes incompetent to make his own decisions.

Thirdly, the possibilities of doing research without the consent of the research participant are rather limited. Proxy consent is a less robust authorization than authorization by the research subject himself, and consent by a legal representative has only little moral authority compared to autonomous authorization by the research subject. Therefore, the measure and extent to which a third party may expose the incompetent research subject to risk or harm is limited to either therapeutic research, or non-therapeutic research with minimal risk and minimal burden (e.g. Biomedicine Convention 1997; European Clinical Trials Directive 2001). The possibilities of doing research with dementia patients, based on proxy consent, are therefore limited.

## Advance research directives as a reasonable option

Given the often slowly progressive nature of dementia, and therefore the possibility of anticipating future incompetence, dementia patients could anticipate future research participation by signing an ARD. Dementia patients have “a history of autonomy” and have lived a life in which they have expressed their ideals and preferences. Now they have lost the capacities to make their own decisions, their own voice should still matter in the authorization process. Advance directives make this possible to some extent. We will argue that (1) autonomous decisions may be directed at the future (2) that respecting autonomy goes beyond respecting the wishes of competent persons, (3) that ARDs capture autonomous wishes and (4) legal representatives may benefit from ARDs.

Firstly, if the principle of respect for autonomy requires us to respect a competent patient’s decisions, then it also requires us to respect such decisions made in advance. A person can exercise autonomy not only by making decisions in the present, but also by making decisions that will influence what is to happen in the future (Davis 2007). The difference between autonomy and anticipated or so called precedent autonomy is that precedent autonomy involves a longer passage of time, and the mere passage of time makes no difference to the moral authority of an agent’s autonomous act (Rhoden 1990). For many other decisions we reason according to this principle as well; the marriage, living will, advance care directive and the mortgage of the dementia patient remain lawful, even though there has been a passing of time. We have no reason to presume that an autonomous decision concerning research participation should be treated differently.

Secondly, respecting autonomy also implies respecting former decisions that shaped and gave meaning to the life of the now incompetent person (Buchanan and Brock 1990), as long as the decision is not changed or renounced in the meantime. Dementia robs patients of the capacities to understand or reaffirm the prior expressed wishes. Having lost the capacities to reaffirm prior preferences is, however, not the same as having changed or renounced their prior preference. In order to find out whether the prior set preferences are still applicable, we should try to imagine what the dementia patient would prefer in the current situation, if he were competent to decide^5^ (Davis 2002). Think for example about a Jehovah’s witness who has explicitly stated that he does not want to receive a blood donation if he would need it during surgery, this request remains to be an autonomous wish, and remains authoritative and leading, even if the Jehovah’s witness is unconscious and would, from a medical perspective, need a blood transfusion in order to survive. If there are no strong reasons to assume otherwise, the prior stated preferences continue to carry significance and should be the default for decision-making.

Thirdly, an ARD captures the preferences of an autonomous person directed at the future and functions as a means to exercise one’s right to choose a future beyond one’s decision-making capacity (Beauchamp and Childress  2013; Davis 2007; Vollmann 2001; Dworkin 1986; Alzheimer Europe 2006). In an ARD the dementia patient can, at the time that he is still competent, describe his preferences concerning research participation and describe which risks and burden he is willing to bear. Thereby consent with an ARD does justice to the moral aim of autonomous authorization of informed consent, because it is an authorization given by the autonomous research subject. If we consider the authorization given in an ARD similar to authorization of informed consent, a research subject should also be allowed to authorize his participation in research trials containing more than minimal risk and burden (Pierce 2010; Buller 2014), as long as the patient was also informed and free to decide at the moment the ARD was written. An ARD would thus allow for a broader range of research trials than the current legal standard, and could enable valuable research in the search of a treatment for dementia. Besides, making an advance directive is itself an exercise of autonomy. The person may benefit from knowing that he has done everything he could to be treated in the way he wants to at times of incompetence (Singer et al. 1992).

On a more practical level, we recognise that dementia patients are very dependent on their legal representatives, even if only for logistical and practical support. Legal representatives will regardless of the existence of an ARD, remain to play a role when the dementia patient becomes incompetent. ARDs can also help the legal representative in supporting research decisions according to the research participant’s prior preferences.

## Controversies of ARDs

We have argued that there are strong reasons why ARDs provide an ethically permissible base for research authorization; there are also some difficulties that should be considered carefully. In this section we will discuss some concerns and controversies to the use of ARDs for dementia patients.

### The moral authority of the ARD

A general claim against the use of advance directives, is that respect for autonomy should not be primary in decision making for dementia patients, and that the “best-interest” principle should be primary (Dresser 1992; Robertson 1991). This objection to autonomy trumping other values, entails that decisions should be based on choices that are beneficial to the dementia patient according to the assessment of others and it is assumed that (1) preferences do not survive the loss of mental incapacity, or (2) the person is incompetent to decide about future questions, therefore autonomy should not be primary.

The first assumption implies that preferences formulated while competent no longer have meaning once the person is incompetent. An advance directive that captures prior preferences should therefore not be followed. Instead, it is argued, the patient’s current wishes should be primary in decision-making. It is a questionable assumption to state that the dementia patient’s preferences and values do not survive the cognitive decline, because the cognitive decline implies a loss of functions, not necessarily of values. The difficulty in the case of dementia patients is of course that they may not express the prior expressed preference anymore, but that is not the same as having changed or renounced prior preferences. When cognitive capacities decline, and reflection upon their preferences and values is not possible anymore, we cannot simply argue that this person has developed a new preference or has changed his earlier values. Due to dementia, capacities to remember and live according to prior preferences are forgotten, rather than changed consciously. Furthermore, which values and preferences remain is not the result of a reflective process; therefore we cannot assume that the current unreflected wishes of the incompetent dementia patients should be primary. Dementia patients lose the capacities to make their own decisions, but it is unwarranted to conclude that their life is not their own anymore, and that their own earlier preferences no longer matter.

The second assumption regards the incompetence to decide about future decisions. In general we agree to respect decisions of competent persons in the research context, when they are free from coercion and based on sufficient information, even if these decisions might harm the person in question. The sufficiency of information can be questioned when during the time gap between signing and the use of the ARD, new information about the research trial or about specific procedures emerges. Authorization of research participation should ideally be based on the most up-to-date information in order to inform the research participant adequately. The moral authority of an ARD based on insufficient or false information is weakened (Buchanan and Brock 1990; Davis 2007). However, this does not mean that an ARD will never be based on sufficient information. As we have argued before, a research participant needs to be informed adequately, which does not imply *fully* informed. Therefore, as long as the anticipated authorization is based on sufficient and up-to-date information, the authorization given with an ARD should be considered a competent and valid decision.

Dresser (1999) states that an anticipated decision cannot be a truly informed decision, because the competent person needs to anticipate a situation he has never experienced; namely being incapacitated. It may be difficult for a healthy person to anticipate one’s own wishes when ill. The future-oriented preference is based on assumptions, that may either overestimate the suffering or underestimate the burden the illness or medical interventions will impose (Dresser 1986). It may be even more difficult to anticipate the ill *and* cognitively impaired persons’ experience of research participation (Dresser 2001, 2014). It is questioned whether any competent person is ever fully able to anticipate the point of view of his incapacitated future version (Dresser 1999; Fagerlin and Schneider 2004). It remains however unclear why from the anticipatory character of the decision, it should follow that prior preferences are to be ignored altogether. To illustrate that we generally accept anticipatory decisions from competent persons, independent of whether they are foreseen accurately or inaccurately, consider the following example. A person might want to have a tattoo, but has no clue whether he will be a person who likes tattoos when he is 65, but he also has no clue whether he will regret it when he has not taken a tattoo in fear of his future judgement. Whether he gets a tattoo or not, as long as he is competent, is a valid and autonomous decision, regardless of whether he agrees with it later or not. The possibility of being mistaken in hindsight, with a former anticipatory decision, is no reason to disregard the moral authority of this decision. It is, however, a good reason to allow dementia patients a way out, when they indeed appear to have foreseen the decision wrongly.

In more general terms, the best-interest account is disanalogous in the research context because research is never primarily in the participants best-interest. It could be argued that therapeutic effects from a research trial are “a reasonable person’s” best interest, but therapeutic effects alone are never a sufficient reason to include anybody in any research study.

Furthermore, the best-interest account overlooks the point that the dementia patient has not always been incompetent to decide. It remains unclear, why any proxy would be in the best position to decide about research participation and how this proxy can decide about the *willingness* to participate. Even if the legal representative would succeed in giving voice to the preferences of the research subject, it remains doubtful that a proxy would be better at estimating what is in the person’s interest than the research participant himself. Furthermore, legal representatives may decide for the now incompetent person, *because* the representative is competent. Deciding for an incompetent person would, according to Dresser (1999) and Fagerlin and Schneider (2004) argument, involve the anticipation to a state of incompetence, which is arguably an even more difficult task for a legal representative, since he would not only have to anticipate to this state of incompetence but also to the point of view of the person they represent. It is therefore a questionable assumption that it is a better option to let the legal representative decide for incompetent research subjects. An ARD provides a more solid moral foundation for decision-making than the best interest account and the current standard of consent by a legal representative.

## Protection during the trial: risk, burden and withdrawal

Regardless of how the consent is given, by a research participant himself or by consent of a proxy, moral and practical questions emerge when the research participant does not want to (continue to) participate when the research takes place. This problem is thus not specific for the use of ARDs, and appears after initial authorization is given. This problem is related to the idea that informed consent should be a continuous process, rather than a momentary authorization, and the willingness to participate should persist during the trial.

At this point, we should make an important and necessary distinction between the anticipation of future risks and the anticipation of future burden. Arguably, the anticipation of risks is more stable than the anticipation of burden due to the nature of these concepts. The assessment whether a risk is acceptable is based on abstract information and depends on characteristics of a procedure. In order to assess whether a person is willing to take a risk, it is necessary to be competent, because information forms the base for risk-assessment. The willingness to take a certain risk does not change over the course of a trial, as long as the provided information has been accurate. By contrast, the assessment of burden has an experiential element, and as dementia patients remain able to have subjective experiences, they are still able to experience the burden (Berghmans 2000). Therefore, even though the burden seemed acceptable when the ARD was signed, the burden might be *experienced* differently. This warrants for extra precautions during the trial. The research participant deserves to be protected against *undue* burden, especially when he is incapacitated. This objection does not question the initial authorization given by an ARD, but shows that withdrawal can be problematic for this population. Moreover it is important to mention that withdrawal from research, in contrast to consent, does not need to be an autonomous decision, and may be done for any reason at any time. However, in order to be able to withdraw from a trial, it is at least necessary to know or remember that you are taking part in a research trial. Research participants suffering from dementia may have forgotten this.

As dementia patients are limited in their abilities to express reasons for withdrawal; they largely depend on others for protecting their well-being during the trial. The level of burden should be monitored continuously and when the participant objects more than anticipated to a research procedure, we have reasons to withdraw him from the procedure, because we cannot be sure the participant is willing to continue participation. Here also lies a role for legal representatives, to act as a safeguard against exploitation and to provide protection.

## What this all implies for Mr. Jansen

Autonomous decision-making is an important corner stone for research participation and, from a moral point of view; we argue that respect for autonomy cannot be disregarded for demented research participants. It is clearly desirable for persons to be able to have some say about their future and extend the influence of their autonomously formed preferences. It is precisely in anticipating circumstances in which one does not have the capacities to make decisions anymore, that dementia patients may want their preferences to be followed, in order to give direction to their own lives. Even though dementia patients might want to be careful with their future selves, that will be more vulnerable and less able to carry burden, the decision of how much burden they are willing to bear and to what extent the current person’s interests may be compromised should remain up to them. Therefore, his precedent autonomy should remain primary, as long as there are no strong reasons to assume otherwise. The ARD is in such cases valid as an initial authorization and is given by an autonomous agent, and would thus allow for the same range of risk and burden in research trials as would informed consent. Thereby ARDs would enable valuable research in the search of a treatment for dementia.

The objections against the use of ARDs (Fagerlin and Schneider 2004; Dresser 1992, 1999, 2014; Robertson 1991) are objections against advance directives in general. ARDs are a special type of advance directive, because they authorize research participation. While for treatment in some cases the will of the patient may be overruled, it is never acceptable to include a research participant in a trial without his consent. Possible therapeutic effects alone are not enough reason to justify research participation. Authorization is necessary, even if it is likely that a trial will benefit the participant; this underlines the difference between research and treatment (Jongsma and van de Vathorst 2014). Furthermore the opponents of advance directives do not convince in disregarding the anticipated preferences of the dementia patients nor do they succeed in disproving the moral authority of ARDs. They merely show that autonomous authorization is necessary, but not sufficient, and we should allow dementia patients a way out, when they are burdened more than anticipated.

Respect for persons implies that we should not only respect prior autonomy, but also protect the no longer autonomous patients *during* the trial. We therefore need to find an adequate balance between respecting the prior autonomy of dementia patients and protecting the incompetent research participant during the trial. Dementia patients remain conscious and present during the trial, as opposed to many other situations advance directives are used for; i.e. post mortem directives. We should therefore remain cautious and look for signs of resistance or objection to undue burden during the research procedures. Any indication that the incapacitated person is suffering more than anticipated should be taken seriously and is a good reason to stop the research procedure, but does not imply the overall exclusion of this person from research altogether. The wish to stop may not based on the person’s values, because it is unreflected and temporary, and should not be understood as an act against the moral authority of the ARD. The temporary resistance against the research procedure should result in stopping the research procedure, but continuation in the research trial at a later period of time, based on the anticipated preference, would be tenable.

Coming back to the case described in the introduction, we would like to argue that it is a lost opportunity that Mr. Jansen has little to say in his current research decisions. Autonomous authorization is required for doing research with human participants. The exclusion of dementia patients from the authorization process altogether is not self-evidently justifiable. From a moral point of view, we argue that there should be more attention to the prior autonomy of demented research participants, particularly as research does not primarily aim to benefit the research participants. An ARD offers Mr. Jansen the opportunity to give direction to his life beyond his own competence. As autonomous persons are considered to be in the best position to give direction to their lives, anticipated autonomous decisions should be respected as well. As long as Mr. Jansen is competent to give informed consent he can, at least in Europe, participate in research trials containing more than minimal risks, also when these trials do not directly benefit him. We have argued that as long as an anticipated authorization is based on sufficient and up-to-date information, it should be allowed to cover the same range of research trials as informed consent of a competent person.

## Conclusion

Consent to participation in research is ideally based on informed, free and competent authorization, by an agent who has “here and now” autonomy. This ideal is out of reach for incapacitated dementia patients, as they are no longer competent. The current legal standard for including incompetent research subjects in research trials requires the consent of the legal representative, but fails to do justice to the moral aim of respect for autonomy. It is remarkable that there is currently only limited attention to the moral aim of autonomous authorization of dementia patients. ARDs offer dementia patients a way to control their life beyond their own competence.

ARDs can help in authorising research participation, but we should provide protection to research subjects once they become incompetent. This protection is necessary during the trial, by remaining cautious of burdening incompetent research participants more than they anticipated. The remaining issues of using advance directives for research subjects with dementia do not question the ARD as an authorization tool, but all have to do with withdrawal and resistance.

We conclude that both aims of respect for persons, authorization and protection, are served when ARDs are used to authorize for research participation. ARDs allow patients to keep control beyond their own incompetence and are a morally defensible basis for authorizing research participation, but ARDs cannot solve all problems of doing research with incapacitated participants, therefore extra precautions remain necessary.

## Footnotes

1 We have not specified due to which disease Mr. Jansen is suffering from dementia, because we aim to describe situations concerning cognitive decline, regardless of the underlying neurodegenerative disease. Dementia is a collective term, but is not a disease itself. When we write a dementia patient, we mean a person suffering from the symptoms of dementia due to an underlying illness, such as Alzheimer’s disease.

2 In Europe, most guidelines and legislation set additional protective rules for the inclusion of participants unable to consent, as consent of a legal representative is a necessary, but not sufficient condition. Additional requirements include a direct benefit to the research subject, or if that is not likely the study should not convey more than a minimal risk. These requirements are formulated to protect the vulnerable research participant.

3 Switzerland is a noteworthy exception, with Art. 24, para. 1 of the domestic law stipulating that “[r]esearch projects involving persons unable to consent which offers prospects of direct benefit to participants can be conducted if the following conditions are met: 1. the subjects have given their consent when they were still competent and the consent is *evidenced by a document*;.” in the law.

4 Autonomy and competence differ in meaning, autonomy implies self-governance and competence implies the ability to perform a task, but the criteria for the competent person and for the autonomous person are remarkably similar (Beauchamp and Childress 2013).

5 Davis (2002) notes that it is impossible to be demented and have full mental capacity at the same time, therefore rather than imagining the patient in her actual circumstances with full mental capacity, we should imagine the patient in circumstances *as similar as possible* to her actual circumstances, but in such a manner that, unlike her actual condition, she has full mental capacity.
